# Numerical Study of Nanofluid Irreversibilities in a Heat Exchanger Used with an Aqueous Medium

**DOI:** 10.3390/e22010086

**Published:** 2020-01-10

**Authors:** Guillermo Efren Ovando-Chacon, Sandy Luz Ovando-Chacon, Abelardo Rodriguez-Leon, Mario Diaz-Gonzalez, Jorge Arturo Hernandez-Zarate, Alberto Servin-Martinez

**Affiliations:** 1 National Technological of Mexico, Veracruz Institute of Technology, Calzada Miguel Ángel de Quevedo 2779, Veracruz, Ver 91860, Mexico; arleonver@gmail.com (A.R.-L.); mrdzgn37@gmail.com (M.D.-G.); jorgeahz677@yahoo.com.mx (J.A.H.-Z.); alservinmm@gmail.com (A.S.-M.); 2 National Technological of Mexico, Technological Institute of Tuxtla Gutierrez, Carretera Panamericana km 1080, Tuxtla Gutierrez, Chis 29000, Mexico; ovansandy@hotmail.com

**Keywords:** nanofluid, heat exchanger, entropy generation, heat transfer, performance evaluation criteria

## Abstract

Heat exchangers play an important role in different industrial processes; therefore, it is important to characterize these devices to improve their efficiency by guaranteeing the efficient use of energy. In this study, we carry out a numerical analysis of flow dynamics, heat transfer, and entropy generation inside a heat exchanger; an aqueous medium used for oil extraction flows through the exchanger. Hot water flows on the shell side; nanoparticles have been added to the water in order to improve heat transfer toward the cold aqueous medium flowing on the tube side. The aqueous medium must reach a certain temperature in order to obtain its oil extraction properties. The analysis is performed for different Richardson numbers (*Ri* = 0.1–10), nanofluid volume fractions (*φ* = 0.00–0.06), and heat exchanger heights (*H* = 0.6–1.0). Results are presented in terms of Nusselt number, total entropy generation, Bejan number, and performance evaluation criterion. Results showed that heat exchanger performance increases with the increase in *Ri* when *Ri* > 1 and when reducing *H*.

## 1. Introduction

Over the past decades, research has sought new ways of improving heat transfer for applications in different fields, such as electronics, petrochemistry, food preparation, communications, etc. Ways to improve heat transfer processes are to add extension surfaces such as fins to the system or to change the geometry of the device to increase contact surface area; however, the capabilities of these techniques are reaching their limit. Because metals in solid form at room temperature have thermal conductivities several orders of magnitude larger than water, one way to increase heat transfer in conventional fluids is to add solid metal particles that increase the thermal conductivity of the fluid. The improvement of thermal properties in suspensions containing solid particles has been studied for more than a century; however, their millimetric size results in flow resistance, erosion, and obstruction issues in narrow channels. Two decades ago, as a result of technological advances in nanotechnology, the Argonne National Laboratory in the United States studied how the use of nanoparticles in fluids could improve the thermal conductivity of the fluid and thus increase heat transfer [1].

Studies and numerical simulations focused on the behavior of nanofluids within devices have been carried out using two approaches. One of them considers that the assumption of a continuous medium is still valid for fluids containing suspended nanoparticles, while the other approach considers two phases as a better description of the phenomenon; between these two, the single-phase approach is computationally simpler and more efficient. Therefore, the present study used the single-phase model and assumed that the medium was continuous. In this regard, several studies on complex fluid [2,3,4,5,6,7] and nanofluid energy transport have been carried out for different configurations, such as microchannels [8,9], corrugated channels [10,11], grooved channels [12,13], circular tubes [14,15], and cavities [16,17,18,19,20,21,22].

In general, most thermal systems are analyzed from the point of view of the first law of thermodynamics, and their design is based on this principle; however, another important consideration is the efficient use of thermal energy. It is, therefore, necessary to study the entropy created by viscous friction and heat transfer in terms of physical and geometric aspects of the device in order to minimize entropy generation and to establish optimal operating conditions to reduce irreversibilities [23,24,25]. Different studies have already focused on nanofluids in different thermal devices with different configurations and operating conditions. Huminic and Huminic [26] studied heat transfer and entropy generation of nanofluids under laminar flow regime in helical heat exchangers; these authors found that heat transfer coefficients, Nusselt number, and heat exchanger effectiveness increased in proportion to nanoparticle volume concentration, while entropy generation due to thermal effects was reduced. Kolsi et al. [27] carried out numerical simulations of convection and entropy caused by the combined effects of convection and thermocapillarity in a cavity containing a nanofluid. The effects of thermocapillary activity and the increase in the volume fraction of nanoparticles resulted in increased entropy. Kolsi et al. [28] reported on numerical simulations nanofluid flow inside a cubic cavity containing adiabatic blocks. At high Rayleigh numbers, entropy generation was dominated by friction. Entropy generated locally by friction increased in proportion to the percentage of the nanofluid volume fraction. Ji et al. [29] determined entropy distribution in the turbulent flow of a nanofluid using numerical methods; their results indicated that there is a Reynolds number in which a maximum value of the performance evaluation criterion is reached; this value is significantly reduced by increasing the volume fraction of the nanofluid. Belhaj and Ben-Beya [30] examined the magneto-hydrodynamic natural convection of a nanofluid in a square cavity heated sinusoidally from below. They determined that increasing Hartmann number reduces heat transfer and entropy generation; on the other hand, the performance evaluation parameter increased when the Hartmann number and the volume fraction of nanofluid increased. Ismael et al. [31] studied entropy generation in a hybrid nanofluid flow within a movable lid cavity heated using a triangular solid. Convective heat transfer increased significantly with the Richardson number, whereas entropy generation increased slightly. Salari et al. [32] analyzed natural convection and entropy generation in a cavity containing two immiscible fluids: air and a water-based nanofluid. High values of volume fraction of nanofluid reduced total entropy generation and increased the Nusselt number. Additionally, high values of Rayleigh numbers increased total entropy generation and the Nusselt number. Kashani et al. [33] performed numerical simulations of the natural convection of a nanofluid in a two-dimensional cavity with corrugated walls. They found that flow, heat transfer, and entropy generation were affected by density variation and nanoparticle dispersion. Mohammadtabar et al. [34] focused on entropy generation in a rectangular cavity with heated walls. Entropy generation increased with the Rayleigh number. By increasing the concentration of nanoparticles and simultaneously moving the position of the heaters, it was possible to increase the Nusselt number and minimize entropy generation. Ting et al. [35] performed numerical simulations of mixed convection nanofluids in a cavity with wavy bottom wall. They found that varying amplitude has a higher effect on both the total entropy generation and average Bejan number than that of the roughness elements.

Based on our literature review, the generation of entropy in a nanofluid flowing in heat exchangers used for the heating of aqueous media for oil extraction has not yet been studied. The purpose of the present study is to characterize different parameters such as the volume fraction of nanofluid, the Richardson number, and the height of the exchanger to determine their effect on heat transfer, performance and entropy generation in heat exchangers under different operating conditions of an aqueous medium used for oil separation. This study is a contribution to our current knowledge of complex heat transfer phenomena occurring in real open systems with complex geometries where three-dimensional simulations are necessary in order to capture all the phenomena taking place inside and to identify the conditions in which these thermal systems operate with minimal entropy generation. The potential benefits of this study are the design and construction of heat exchangers for the food and chemical industry, biochemical processes, as well as the operation of solar thermal collectors to operate with optimal energy consumption.

## 2. Description of the Problem 

**Figure 1:**
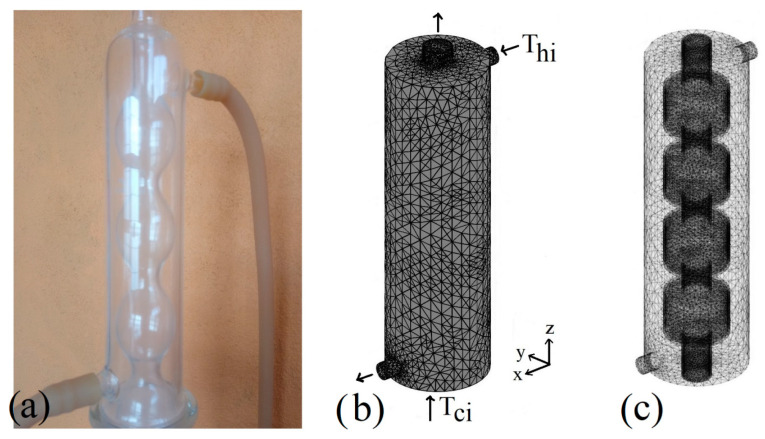
Geometry and mesh of the heat exchanger. (**a**) Actual exchanger; (**b**) exchanger mesh on the enclosure side; (**c**) exchanger mesh on the tube side.

This paper presents a numerical study of irreversibilities within a heat exchanger based on computational fluid dynamics techniques, as shown in Figure 1. This type of heat exchanger is used to maintain an aqueous medium at a correct temperature for oil extraction, where the temperature at which the process is carried out is crucial since it can affect oil extraction performance and oil quality. Simulations are carried out using a three-dimensional geometry. On the shell side, the heating fluid is considered as a nanofluid where different proportions of Al_2_O_3_ metallic nanoparticles are added to the water. On the heat exchanger tube side, the working fluid is considered as an aqueous medium that must be heated by the hotter fluid circulating on the shell side in order to maintain an appropriate temperature that allows for subsequent oil separation. The heating process is achieved by conduction heat transfer through the wall of the heat exchanger tube, which is made of Pyrex glass, as well as by convection heat transfer due to the transport of energy from the heating water on the shell side of the heat exchanger. The analysis is carried out for Richardson *Ri* numbers from 0.1 to 10, for nanofluid volume fractions *φ* from 0 to 0.06, and for different lengths of heat exchanger H from 0.6 to 1. The range of parameters studied in this work corresponds to the aqueous extraction of oil under moderate operating conditions in which oil is obtained at low temperatures (<95 °C). Higher temperatures involve the use of solvents and should be considered a model that includes phase change. It is important to emphasize that the extraction of oil with aqueous media is of great interest today because the extraction with solvents has serious environmental implications. Inlet temperatures on the shell side of the exchanger *θ_hi_* = 1 and on the tube side *θ_ci_* = 0 are used as boundary conditions. Zero temperature gradients are considered for the heat exchanger outlets. Adiabatic temperature conditions are considered for the rest of the external walls of the heat exchanger.

## 3. Conservation Equations 

A system of coupled non-linear equations must be solved to calculate the movement of a non-isothermal incompressible nanofluid with flotation forces in a three-dimensional domain. These are the equations of continuity, momentum, and energy. Using the following dimensionless variables:

(1) $$\begin{array}{ccc} X\;=\;\frac{x}{H_{e}}, & Y\;=\;\frac{y}{H_{e}}, & Z\;=\;\frac{z}{H_{e}},\\ U\;=\;\frac{u}{U_{in}}, & V\;=\;\frac{v}{U_{in}}, & W\;=\;\frac{w}{U_{in}},\\ \tau \;=\;\frac{u_{in}\;t}{H_{e}}, & P\;=\;\frac{p}{\rho U_{in}^{2}}, & \theta \;=\;\frac{T-T_{ci}}{T_{hi}-T_{ci}}, \end{array}$$

where *X* and *Y* are the dimensionless transverse coordinates, *Z* is the dimensionless axial coordinate, *x* and *y* are the transverse coordinates, *z* is the axial coordinate, *U* and *V* are the dimensionless transverse components of velocity, *W* is the dimensionless axial component of velocity, *u* and *v* are the transverse components of velocity, *w* is the axial component of velocity, *τ* is the dimensionless time, t is the time, *P* is the pressure parameter, *p* is the pressure, *θ* is the dimensionless temperature, *T* is the temperature, *H_e_* is the height of the heat exchanger, *U_i_*_n_ is the heating nanofluid inflow velocity on the shell side, *ρ* is the density, *T_ci_* is the temperature of the cold fluid at the inlet, and *T_hi_* is the temperature of the hot fluid at the inlet; in their dimensionless form, conservation equations can be written as follows:

(2) $$\frac{\partial U}{\partial X}+\frac{\partial V}{\partial Y}+\frac{\partial W}{\partial Z}\;=\;0,$$

(3) $$\frac{\partial U}{\partial \tau }+U\frac{\partial U}{\partial X}+V\frac{\partial U}{\partial Y}+W\frac{\partial U}{\partial Z}\;=\;\frac{\rho_{f}}{\rho_{i}}\left[-\frac{\partial P}{\partial X}+\frac{\mu_{i}}{\mu_{f}}\frac{1}{Re}\left(\frac{\partial^{2}U}{\partial X^{2}}+\frac{\partial^{2}U}{\partial Y^{2}}+\frac{\partial^{2}U}{\partial Z^{2}}\right)\right],$$

(4) $$\frac{\partial V}{\partial \tau }+U\frac{\partial V}{\partial X}+V\frac{\partial V}{\partial Y}+W\frac{\partial V}{\partial Z}\;=\;\frac{\rho_{f}}{\rho_{i}}\left[-\frac{\partial P}{\partial Y}+\frac{\mu_{i}}{\mu_{f}}\frac{1}{Re}\left(\frac{\partial^{2}V}{\partial X^{2}}+\frac{\partial^{2}V}{\partial Y^{2}}+\frac{\partial^{2}V}{\partial Z^{2}}\right)\right],$$

(5) $$\frac{\partial W}{\partial \tau }+U\frac{\partial W}{\partial X}+V\frac{\partial W}{\partial Y}+W\frac{\partial W}{\partial Z}\;=\;\frac{\rho_{f}}{\rho_{i}}\left[-\frac{\partial P}{\partial Z}+\frac{\mu_{i}}{\mu_{f}}\frac{1}{Re}\left(\frac{\partial^{2}W}{\partial X^{2}}+\frac{\partial^{2}W}{\partial Y^{2}}+\frac{\partial^{2}W}{\partial Z^{2}}\right)+Ri\frac{{\left(\rho \beta \right)}_{i}}{{\left(\rho \beta \right)}_{f}}\theta \right],\;$$

(6) $$\frac{\partial \theta }{\partial \tau }+U\frac{\partial \theta }{\partial X}+V\frac{\partial \theta }{\partial Y}+W\frac{\partial \theta }{\partial Z}\;=\;\frac{1}{RePr}\frac{\alpha_{i}}{\alpha_{f}}\left(\frac{\partial^{2}\theta }{\partial X^{2}}+\frac{\partial^{2}\theta }{\partial Y^{2}}+\frac{\partial^{2}\theta }{\partial Z^{2}}\right),$$

where *μ_i_*, *ρ_i_*, *α_i_*, and *β_i_* represent dynamic viscosity, density, thermal diffusivity, and coefficient of thermal expansion of the fluid *i*; on the shell side, the working fluid is a nanofluid, and on the tube side, and the fluid is the aqueous medium. The properties of the nanofluid (aluminum oxide) used in the conservation equations on the shell side are *μ_nf_*, *ρ_nf_*, *α_nf_*, and *β_nf_*, which correspond to dynamic viscosity, density, thermal diffusivity, and thermal expansion coefficient, respectively. The properties of the aqueous medium used in the conservation equations on the tube side are *μ_f_*, *ρ_f_*, *α_f_*, and *β_f_*, which correspond to dynamic viscosity, density, thermal diffusivity, and thermal expansion coefficient, respectively. The dimensionlessness of the conservation equations results in the following parameters: Reynolds number, Richardson number, and Prandtl number, which are defined as follows:

(7) $$Re\;=\;\frac{\rho U_{in}H_{e}}{\mu },\;\;\;\;\;\;\;\;\;Ri\;=\;\frac{g\beta \left(T_{hi}-T_{ci}\right)H_{e}}{U_{in}{}^{2}},\;\;\;\;\;Pr\;=\;\frac{\mu }{\rho \alpha },$$

where *g* is the acceleration of gravity. *α_nf_* and *α_f_* can be calculated as follows:

(8) $$\alpha_{nf}\;=\;\frac{k_{nf}}{{\left(\rho C_{p}\right)}_{nf}},\;\;\;\;\;\alpha_{f}\;=\;\frac{k_{f}}{{\left(\rho C_{p}\right)}_{f}},$$

where *k* is the thermal conductivity and *ρC_p_* is the heat capacitance.

Several expressions presented by the literature [36,37] can be used to calculate the effective properties of the Al_2_O_3_-water nanofluid in terms of the volume fraction of the nanofluid *φ*. Nanofluid density is determined by:

(9) $$\rho_{nf}\;=\;\rho_{f}\left(1-\varphi \right)+\varphi \rho_{sol},$$

Nanofluid effective viscosity is calculated as:

(10) $$\mu_{nf}\;=\;\frac{\mu_{f}}{{\left(1-\varphi \right)}^{2.5}},$$

The following expression is used to calculate specific heat:

(11) $${\left(\rho C_{p}\right)}_{nf}\;=\;{\left(\rho C_{p}\right)}_{f}\left(1-\varphi \right)+\varphi {\left(\rho C_{p}\right)}_{sol},$$

Nanofluid thermal conductivity is determined by [36,37]:

(12) $$k_{nf}\;=\;k_{f}\left[\frac{k_{sol}+2k_{f}-2\varphi \left(k_{f}-k_{sol}\right)}{k_{sol}+2k_{f}+\varphi \left(k_{f}-k_{sol}\right)}\right],$$

The following expression is used to calculate the nanofluid thermal expansion coefficient:

(13) $${\left(\rho \beta \right)}_{nf}\;=\;{\left(\rho \beta \right)}_{f}\left(1-\varphi \right)+\varphi {\left(\rho \beta \right)}_{sol},$$

The local Nusselt number on the outer surface on the tube side of the heat exchanger can be determined as [33,38,39,40]:

(14) $${Nu_{l}\;=\;-\left(\frac{k_{nf}}{k_{f}}\right)\frac{\partial \theta }{\partial n}|}_{wall},$$

where *η* is the normal direction. The average Nusselt number on the outer surface on the tube side of the heat exchanger can be calculated as:

(15) $$Nu\;=\;\frac{1}{A}\int^{\;}Nu_{l}\;dA.$$

where *A* is the outer surface area.

## 4. Entropy Generation Equations 

The dimensionless local entropy generation due to heat transfer can be expressed as:

(16) $$S_{lh}\;=\;\frac{k_{nf}}{k_{f}}\left[{\left(\frac{\partial \theta }{\partial X}\right)}^{2}+{\left(\frac{\partial \theta }{\partial Y}\right)}^{2}+{\left(\frac{\partial \theta }{\partial Z}\right)}^{2}\right].$$

The generation of dimensionless local entropy due to fluid friction is given by:

(17) $$S_{lf}\;=\;\emptyset \frac{\mu_{nf}}{\mu_{f}}\left[2{\left(\frac{\partial U}{\partial X}\right)}^{2}+2{\left(\frac{\partial V}{\partial Y}\right)}^{2}+2{\left(\frac{\partial w}{\partial Z}\right)}^{2}+{\left(\frac{\partial U}{\partial Y}+\frac{\partial V}{\partial X}\right)}^{2}+{\left(\frac{\partial U}{\partial Z}+\frac{\partial W}{\partial X}\right)}^{2}+{\left(\frac{\partial V}{\partial Z}+\frac{\partial W}{\partial Y}\right)}^{2}\right].$$

The previous equations determine dimensionless local entropy generation [32,39,40,41]:

(18) $$S_{l}\;=\;S_{lh}+S_{lf}.$$

Total dimensionless entropy generation is obtained by integrating Equation (18) into the computational domain *υ*:

(19) $$S\;=\;\frac{1}{\upsilon }\int^{\;}S_{l}d\upsilon .$$

Another important parameter to determine the importance of irreversibilities due to heat transfer is the Bejan number:

(20) $$Be\;=\;\frac{S_{lh}}{S_{l}}.$$

If *Be* = 1/2, entropy generation due to viscous effects and heat transfer are equal. Entropy generation due to fluid friction is the most important source of entropy when *Be* < 1/2, whereas when *Be* > 1/2, heat transfer is the main entropy generator.

## 5. Numerical Methods

The computational method used to solve the conservation equations in the present study is based on the finite element method, see Glowinsky [42] and Girault and Raviart [43]. The operator splitting scheme is used to decouple non-linearity from the Navier–Stokes equation. This technique consists in separating the conservation equations in the following subproblems:

(21) $$\int_{\Omega }^{}\frac{U^{n+\frac{1}{3}}-U^{n}}{\Delta \tau }\psi d\Omega +\frac{\rho_{f}}{\rho_{i}}\int_{\Omega }^{}P^{n}\frac{\partial \psi }{\partial X}d\Omega \;=\;0,$$

(22) $$\int_{\Omega }^{}\frac{V^{n+\frac{1}{3}}-V^{n}}{\Delta \tau }\psi d\Omega +\frac{\rho_{f}}{\rho_{i}}\int_{\Omega }^{}P^{n}\frac{\partial \psi }{\partial Y}d\Omega \;=\;0,$$

(23) $$\int_{\Omega }^{}\frac{W^{n+\frac{1}{3}}-W^{n}}{\Delta \tau }\psi d\Omega +\frac{\rho_{f}}{\rho_{i}}\int_{\Omega }^{}P^{n}\frac{\partial \psi }{\partial Z}d\Omega \;=\;0,$$

(24) $$\int_{\Omega }^{}\left(\frac{\partial U^{n+\frac{1}{3}}}{\partial X}+\frac{\partial V^{n+\frac{1}{3}}}{\partial Y}+\frac{\partial W^{n+\frac{1}{3}}}{\partial Z}\right)\psi d\Omega \;=\;0,$$

(25) $$\int_{\Omega }^{}\frac{U^{n+\frac{2}{3}}-U^{n+\frac{1}{3}}}{\Delta \tau }\psi d\Omega +\int_{\Omega }^{}\left(U^{n+\frac{1}{3}}\frac{\partial U^{n+\frac{2}{3}}}{\partial X}+V^{n+\frac{1}{3}}\frac{\partial U^{n+\frac{2}{3}}}{\partial Y}+W^{n+\frac{1}{3}}\frac{\partial U^{n+\frac{2}{3}}}{\partial Z}\right)\psi d\Omega \;=\;0,$$

(26) $$\int_{\Omega }^{}\frac{V^{n+\frac{2}{3}}-V^{n+\frac{1}{3}}}{\Delta \tau }\psi d\Omega +\int_{\Omega }^{}\left(U^{n+\frac{1}{3}}\frac{\partial V^{n+\frac{2}{3}}}{\partial X}+V^{n+\frac{1}{3}}\frac{\partial V^{n+\frac{2}{3}}}{\partial Y}+W^{n+\frac{1}{3}}\frac{\partial V^{n+\frac{2}{3}}}{\partial Z}\right)\psi d\Omega \;=\;0,$$

(27) $$\int_{\Omega }^{}\frac{W^{n+\frac{2}{3}}-W^{n+\frac{1}{3}}}{\Delta \tau }\psi d\Omega +\int_{\Omega }^{}\left(U^{n+\frac{1}{3}}\frac{\partial W^{n+\frac{2}{3}}}{\partial X}+V^{n+\frac{1}{3}}\frac{\partial W^{n+\frac{2}{3}}}{\partial Y}+W^{n+\frac{1}{3}}\frac{\partial W^{n+\frac{2}{3}}}{\partial Z}\right)\psi d\Omega \;=\;0,$$

(28) $$\int_{\Omega }^{}\frac{\theta^{n+\frac{2}{3}}-\theta^{n}}{\Delta \tau }\psi d\Omega +\int_{\Omega }^{}\left(U^{n+\frac{1}{3}}\frac{\partial \theta^{n+\frac{2}{3}}}{\partial X}+V^{n+\frac{1}{3}}\frac{\partial \theta^{n+\frac{2}{3}}}{\partial Y}+W^{n+\frac{1}{3}}\frac{\partial \theta^{n+\frac{2}{3}}}{\partial Z}\right)\psi d\Omega \;=\;0,.\;$$

(29) $$\int_{\Omega }^{}\frac{\theta^{n+1}-\theta^{n+\frac{2}{3}}}{\Delta \tau }\psi d\Omega +\frac{1}{RePr}\frac{\alpha_{i}}{\alpha_{f}}\int_{\Omega }^{}\left(\frac{\partial \theta^{n+1}}{\partial X}\frac{\partial \psi }{\partial X}+\frac{\partial \theta^{n+1}}{\partial Y}\frac{\partial \psi }{\partial Y}+\frac{\partial \theta^{n+1}}{\partial Z}\frac{\partial \psi }{\partial Z}\right)d\Omega \;=\;\int_{\Gamma }^{}\theta_{D}\psi d\Gamma ,$$

(30) $$\int_{\Omega }^{}\frac{U^{n+1}-U^{n+\frac{2}{3}}}{\Delta \tau }\psi d\Omega +\frac{\rho_{f}}{\rho_{i}}\frac{\mu_{i}}{\mu_{f}}\frac{1}{Re}\int_{\Omega }^{}\left(\frac{\partial U^{n+1}}{\partial X}\frac{\partial \psi }{\partial X}+\frac{\partial U^{n+1}}{\partial Y}\frac{\partial \psi }{\partial Y}+\frac{\partial U^{n+1}}{\partial Z}\frac{\partial \psi }{\partial Z}\right)d\Omega \;=\;\int_{\Gamma }^{}U_{D}\psi d\Gamma ,$$

(31) $$\int_{\Omega }^{}\frac{V^{n+1}-V^{n+\frac{2}{3}}}{\Delta \tau }\psi d\Omega +\frac{\rho_{f}}{\rho_{i}}\frac{\mu_{i}}{\mu_{f}}\frac{1}{Re}\int_{\Omega }^{}\left(\frac{\partial V^{n+1}}{\partial X}\frac{\partial \psi }{\partial X}+\frac{\partial V^{n+1}}{\partial Y}\frac{\partial \psi }{\partial Y}+\frac{\partial W^{n+1}}{\partial Z}\frac{\partial \psi }{\partial Z}\right)d\Omega \;=\;\int_{\Gamma }^{}V_{D}\psi d\Gamma ,$$

(32) $$\int_{\Omega }^{}\frac{W^{n+1}-W^{n+\frac{2}{3}}}{\Delta \tau }\psi d\text{Ω}+\frac{\rho_{f}}{\rho_{i}}\frac{\mu_{i}}{\mu_{f}}\frac{1}{Re}\int_{\text{Ω}}^{}\left(\frac{\partial W^{n+1}}{\partial X}\frac{\partial \psi }{\partial X}+\frac{\partial W^{n+1}}{\partial Y}\frac{\partial \psi }{\partial Y}+\frac{\partial W^{n+1}}{\partial Z}\frac{\partial \psi }{\partial Z}\right)d\text{Ω}+\int_{\text{Γ}}^{}W_{D}\psi d\text{Γ}.$$

The new field of temperature, velocities, and pressure are obtained by solving these subproblems. The procedure is repeated until the permanent state is reached when the consecutive values of each variable are less than 10^−6^.

In order to guarantee that the computed results are accurate, five different meshes are tested. The convergence of the numbers of Nussselt, entropy, and Bejan are shown in Table 1, for two different cases with different meshes. The mesh-independence study indicates that a mesh of 350,422 nodes guaranteed a reliable solution (less than 1% difference), so this mesh is used in the simulations.

## 6. Validation 

**Figure 2:**
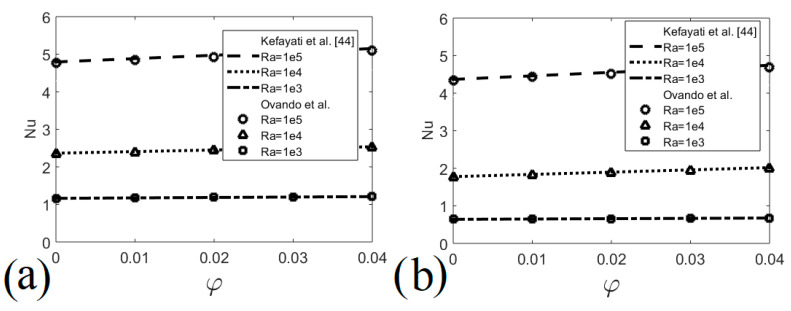
Nu validation of the current code. (**a**) Cavity with aspect ratio 1; (**b**) Cavity with aspect ratio 2.

**Figure 3:**
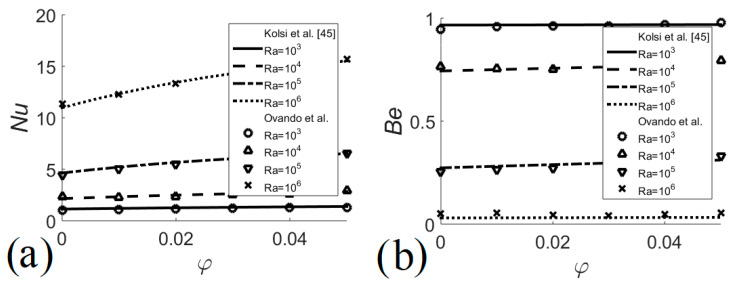
Heat transfer and irreversibilities validation of the current code. (**a**) Nusselt number; (**b**) Bejan number.

The numerical code used in the present study has been validated with different nanofluid problems presented by other researchers. Figure 2 shows comparisons of Nusselt numbers as a function of the volume fraction of nanofluid obtained by the present code and results by Kefayati et al. [44] for different Rayleigh numbers and two different aspect ratios with a maximum difference of 2.3%. Figure 3 presents comparisons of Nusselt number and Bejan number as a function of the nanofluid volume fraction obtained by the present code and results by Kolsi et al. [45] for different Rayleigh numbers, the maximum difference being 2.8%. In addition, temperature measurements were made in the upper, middle and lower parts of the outer surface of the heat exchanger, observing a maximum difference of 1.27% between the measured temperatures and those calculated numerically, see Table 2.

## 7. Results and Discussion

This section presents results and analyzes the parameters that quantify heat transfer, irreversibilities, and heat exchanger performance in terms of Nusselt number, entropy and performance evaluation criterion as a function of nanofluid volume fraction, Richards on number, and heat exchanger size.

### 7.1. Heat Transfer

**Figure 4:**
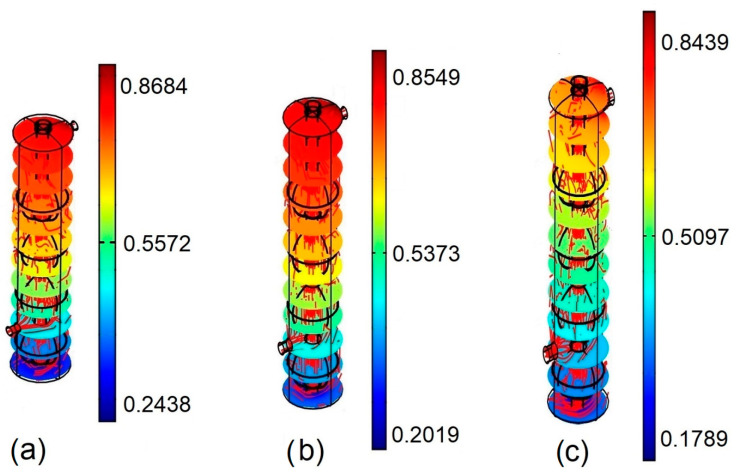
Temperature field for *Ri* = 1, *φ* = 0.02. (**a**) *H* = 0.6; (**b**) *H* = 0.8; (**c**) *H* = 1.0.

The temperature fields (*Ri* = 1, *φ* = 0.02) in Figure 4 show the decrease in heating nanofluid temperature on the shell side as nanofluid flows from top to bottom. The longer the heat exchanger, the lower the temperature of the heating fluid, that is, heat transfer from the hot nanofluid on the shell side toward the cold aqueous medium on the tube side was better for larger heat exchangers. Figure 4a shows the temperature field for a heat exchanger height of *H* = 0.6; as can be observed, heating nanofluid reached a temperature of 0.2438 in the lower part of the shell side of the heat exchanger, while the temperature of the heating nanofluid in the upper part was 0.8684. When the size of the heat exchanger increased to *H* = 0.8, see Figure 4b, the hot nanofluid on the shell side transferred more heat to the cold aqueous medium than in the previous case and the heating nanofluid reached a temperature of 0.2019 at the base and a temperature of 0.8549 at the top. If the size of the heat exchanger was increased further to *H* = 1.0, see Figure 4c, the heat transfer was maximum for this case. The hot nanofluid on the shell side gave off enough heat in such a way that the temperature of the heating nanofluid around the base of the heat exchanger was 0.1789; the same effect was observed in the upper part of the heat exchanger, where the heating nanofluid in the vicinity of the inlet reached a temperature of 0.8439.

**Figure 5:**
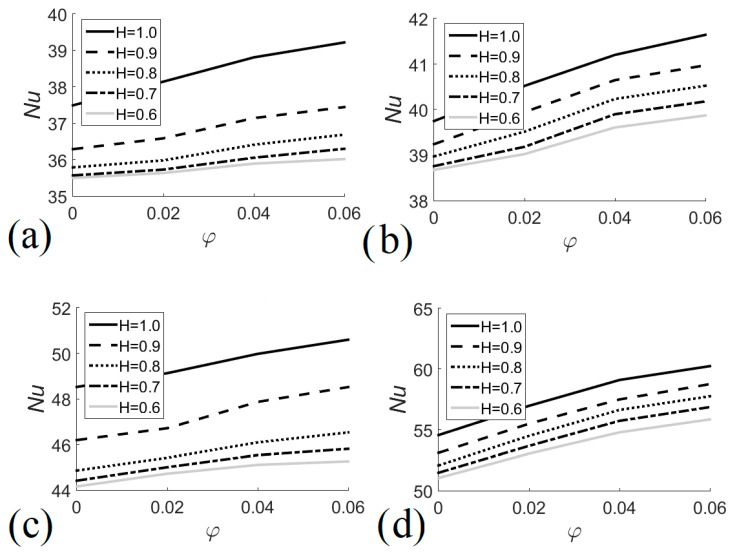
Nusselt number as a function of nanofluid volume fraction for different heat exchanger sizes. (**a**) *Ri* = 0.1; (**b**) *Ri* = 1.0; (**c**) *Ri* = 5.0; (**d**) *Ri* = 10.

The Nusselt number is the parameter that indicates the increase of heat transfer from a surface in contact with a flowing fluid. Figure 5 shows the behavior of the Nusselt number on the outer surface of the tube side of the heat exchanger, where heating nanofluid flows, as a function of the volume fraction of the nanofluid for different Richardson numbers and different sizes of the heat exchanger. In general, the Nusselt number increased with the volume fraction of the nanofluid because thermal conductivity increased when metallic nanoparticles were added to the fluid. On the other hand, the Nusselt number increased with the Richardson number due to the effect of natural convection. Increasing the size of the heat exchanger also increased the Nusselt number due to the increase of the tube’s surface area. The greatest increase in the Nusselt number is due to the increase in the size of the heat exchanger, although the use of a larger exchanger could be impractical since a new heat exchanger would have to be manufactured or purchased; as an alternative, nanofluids could be used as a heating medium to improve natural convection inside an existing heat exchanger. For *Ri* = 0.1, the minimum and maximum Nu values were 35.49 and 39.22 for *H* = 0.6 with *φ* = 0 and *H* = 1.0 with *φ* = 0.06, respectively, see Figure 5a. In the case of *Ri* = 1.0, Figure 5b shows that the minimum and maximum values were 38.67 and 41.64 for *H* = 0.6 with *φ* = 0 and *H* = 1.0 with *φ* = 0.06, respectively. Figure 5c shows that the minimum and maximum values obtained with *Ri* = 5 were 44.16 and 50.59 for *H* = 0.6 with *φ* = 0 and *H* = 1.0 with *φ* = 0.06, respectively. For *Ri* = 10, the minimum and maximum Nu values were 51.04 and 60.24 for *H* = 0.6 with *φ* = 0 and *H* = 1.0 with *φ* = 0.06, respectively, see Figure 5d.

**Figure 6:**
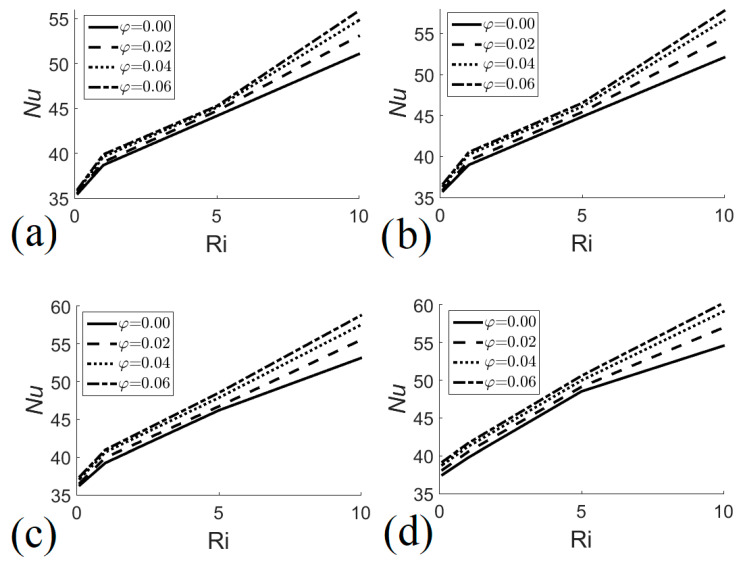
Nusselt number as a function of Richardson number for different nanofluid volume fractions. (**a**) *H* = 0.6;(**b**) *H* = 0.8; (**c**) *H* = 0.9; (**d**) *H* = 1.0.

Figure 6 presents the Nusselt number as a function of the Richardson number for different nanofluid volume fractions and different heat exchanger heights. The graphs confirm that the Nusselt number increases with the Richardson number, the nanofluid volume fraction, and the size of the heat exchanger. Figure 6a shows that the minimum and maximum values of the Nusselt number are 35.49 and 55.85 for *H* = 0.6, and that they are obtained when *φ* = 0 with *Ri* = 0.1 and *φ* = 0.06 with *Ri* = 10, respectively. Figure 6b indicates that the minimum and maximum values of the Nusselt number are 35.78 and 57.76 for *H* = 0.8, obtained when *φ* = 0 with *Ri* = 0.1 and *φ* = 0.06 with *Ri* = 10, respectively. For *H* = 0.9, the minimum and maximum values of the Nusselt number are 36.28 and 58.76, obtained when *φ* = 0 with *Ri* = 0.1 and *φ* = 0.06 with *Ri* = 10, respectively, see Figure 6c. For *H* = 1.0, the minimum and maximum values of the Nusselt number are 37.49 and 60.24, obtained when *φ* = 0 with *Ri* = 0.1 and *φ* = 0.06 with *Ri* = 10, respectively, see Figure 6d.

### 7.2. Entropy Analysis

**Figure 7:**
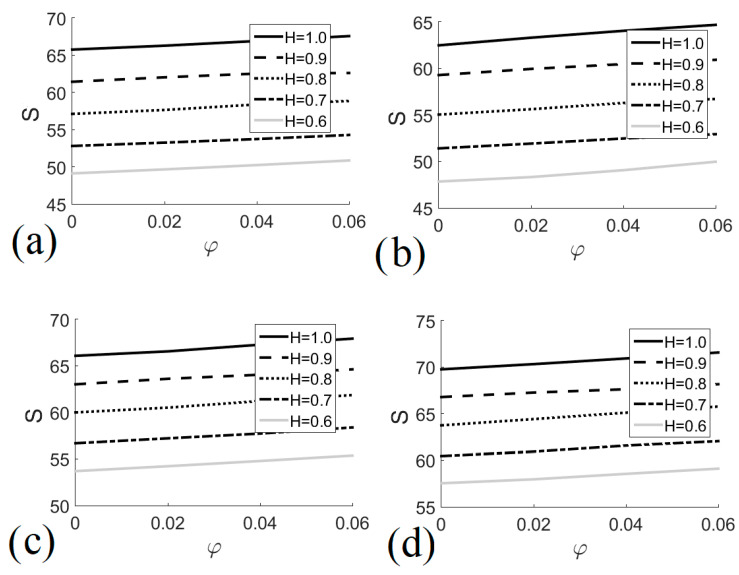
Total entropy generation as a function of nanofluid volume fraction for different heat exchanger sizes. (**a**) *Ri* = 0.1; (**b**) *Ri* = 1.0; (**c**) *Ri* = 5.0; (**d**) *Ri* = 10.

Figure 7 describes the behavior of total entropy generation in terms of nanofluid volume fraction for different heat exchanger heights and different Richardson numbers. In general, irreversibilities increased as the concentration of nanoparticles in the fluid increased, and the size of the heat exchanger increased. System irreversibilities were reduced when *Ri* ≈ 1, that is, when natural and forced convection regimes were of almost equal importance. Figure 7a shows total entropy generation for *Ri* = 0.1; the minimum entropy generation value was 49.11 for *φ* = 0 and *H* = 0.6, whereas the maximum entropy (67.57) occurred when *φ* = 0.06 and *H* = 0.6. In the case of *Ri* = 1.0, Figure 7b shows that the minimum and maximum entropy generation values were 47.84 and 64.69 for *H* = 0.6 with *φ* = 0 and *H* = 1.0 with *φ* = 0.06, respectively. Figure 7c shows total entropy generation for *Ri* = 5.0; the minimum entropy generation value was 53.70 for *φ* = 0 and *H* = 0.6, whereas the highest entropy generation (67.93) occurred when *φ* = 0.06 and *H* = 0.6. Figure 7d shows that the minimum and maximum entropy generation values for *Ri* = 10 were 57.54 and 71.58 for *H* = 0.6 with *φ* = 0 and *H* = 1.0 with *φ* = 0.06, respectively.

**Figure 8:**
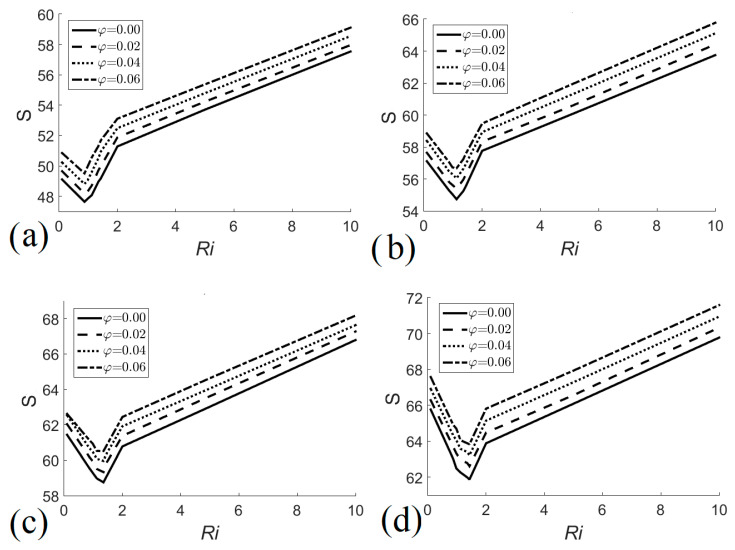
Generation of total entropy as a function of Richardson number for different nanofluid volume fractions. (**a**) *H* = 0.6; (**b**) *H* = 0.8; (**c**) *H* = 0.9; (**d**) *H* = 1.0.

Total entropy generation in the heat exchanger as a function of the Richardson number is presented in Figure 8. An initial decrease in entropy generation with the increase in *Ri* can be observed for the different heights of the heat exchanger and the different nanofluid volume fractions; this trend continues until an optimal value is reached, which occurs around *Ri* ≈ 1.0, that is, close to the region where natural and forced convection regimes are equally important. Subsequently, system irreversibilities increase with the increase in *Ri*. On the other hand, the generation of entropy is increased by increasing the volume fraction of the nanofluid, due to the increase in fluid friction and heat transfer which generates higher velocity and temperature gradients. Figure 8a shows total irreversibility generation as a function of *Ri* for *H* = 0.6; in this case, the minimum entropy generation occurs when *Ri* = 0.87. For *H* = 0.8, Figure 8b shows that the minimum entropy generation values occur when *Ri* = 1.12 for the different nanofluid volume fractions. Figure 8c shows total irreversibility generation as a function of *Ri* for *H* = 0.9; in this case, the minimum entropy generation occurs when *Ri* = 1.35. For *H* = 1.0, Figure 8d shows that the minimum entropy generation values occur when *Ri* = 1.43 for the different nanofluid volume fractions. Entropy is minimized when *Ri* = 1, because for *Ri* < 1 the movement of the fluid tends to increase due to forced convection which increases irreversibilities, i.e., inertial effects and shear stresses are responsible for increasing the generation of entropy in forced convection regime. On the other hand, for *Ri* > 1 the mobility of the fluid tends to increase due to natural convection, which increases the irreversibilities, in this case not only high velocity gradients are present but also high temperature gradients arise through the walls of the heat exchanger on the tube side, so that the generation of entropy in the natural convection regime is greater than that generated in forced convection.

(33) $$PEC\;=\;\frac{Nu}{S}$$

**Figure 9:**
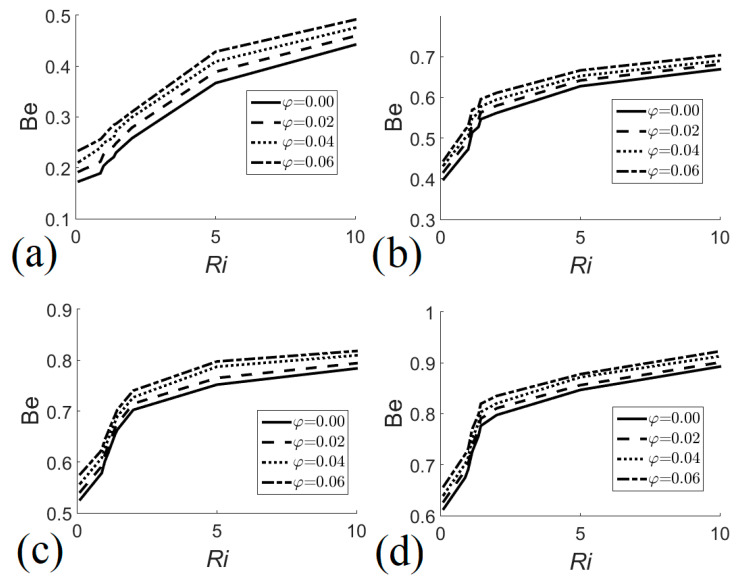
Bejan number as a function of Richardson number for different nanofluid volume fractions. (**a**) *H* = 0.6; (**b**) *H* = 0.8; (**c**) *H* = 0.9; (**d**) *H* = 1.0.

The Bejan number is used to evaluate the importance of entropy generation due to heat transfer, which is defined as the relationship of irreversibility due to thermal effects with total system irreversibility. Figure 9 shows that, in general, the Bejan number increased with the increase of the Richardson number, the nanofluid volume fraction, and the height of the heat exchanger. Figure 9a shows that irreversibilities due to viscous effects were more important when the heat exchanger had a height *H* = 0.6. When the height of the heat exchanger increased to *H* = 0.8, see Figure 9b, irreversibilities due to heat transfer became important if *Ri* > 1. Figure 9c,d show the behavior of the Bejan number for *H* = 0.9 and *H* = 1.0, respectively, revealing that irreversibilities due to thermal effects were predominant in both cases.

### 7.3. Performance Evaluation Criterion

Thus far, the present paper has described heat transfer using the Nusselt number, which is associated with the first law of thermodynamics, and irreversibilities due to non-isothermal flow have been studied in connection with entropy generation, which is associated with the second law of thermodynamics. The combined effect of these two laws of thermodynamics is quantified by the performance evaluation criterion, defined by:

(34) $$PEC=\frac{Nu}{S}$$

**Figure 10:**
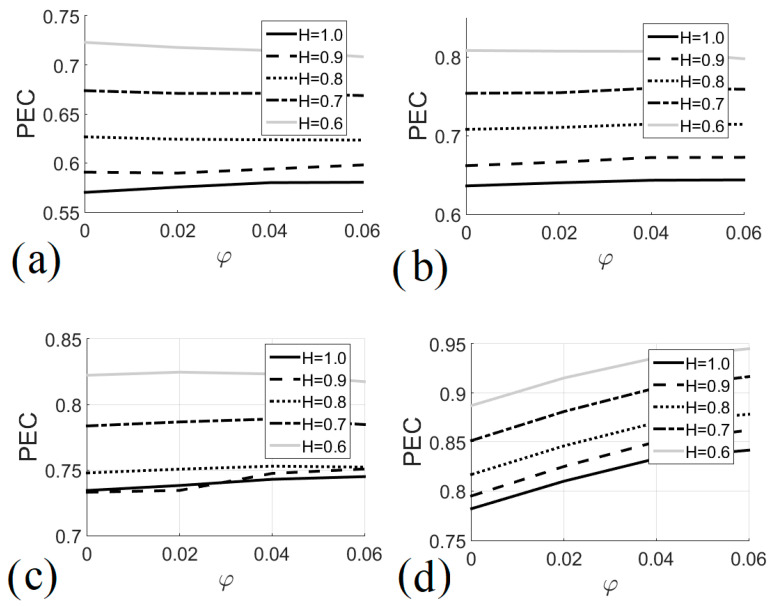
Performance evaluation criterion as a function of nanofluid volume fraction for different heat exchanger sizes. (**a**) *Ri* = 0.1; (**b**) *Ri* = 1.0; (**c**) *Ri* = 5.0; (**d**) *Ri* = 10.

**Figure 11:**
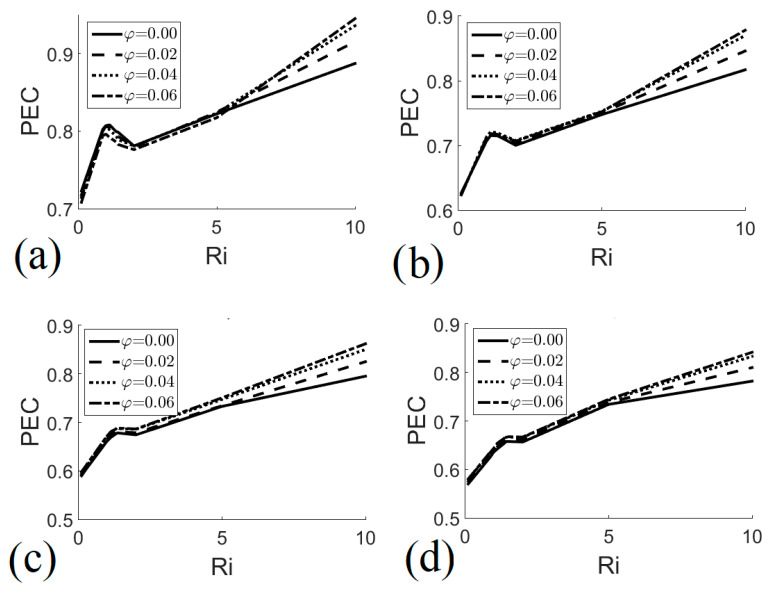
Performance evaluation criterion as a function of Richardson number for different nanofluid volume fractions. (**a**) *H* = 0.6; (**b**) *H* = 0.8; (**c**) *H* = 0.9; (**d**) *H* = 1.0.

Figure 10 shows the higher performance of the heat exchanger in the natural convection regime, i.e., *Ri* = 10 (see Figure 10d); in this case, PEC increased by adding nanoparticles to the working fluid. The lowest performance evaluation criterion values occurred in the forced convection regime, i.e., *Ri* = 0.1 (see Figure 10a); in this case, increasing the number of nanoparticles had no beneficial effect on performance for *H* ≤ 0.8. Figure 11 shows the performance evaluation criterion as a function of the Richardson number for different heat exchanger heights and different nanofluid volume fractions. For all heat exchanger heights, increasing the volume fraction of the nanofluid had no significant effect on performance when 1 < *Ri* < 5; But, when *Ri* > 5, adding nanoparticles improved the performance of the heat exchanger. The general behavior of the PEC as a function of *Ri* was associated with the behavior of Nu and S; in this sense a maximum local value was observed, which occurred when *Ri* ≈ 1; this indicates that heat exchangers that operate with values of *Ri* ≈ 1 for *H* ≥ 0.8 and *φ* = 0.06 should be selected, see Figure 11b–d, which guarantees an optimal heat transfer with the minimum entropy generation and therefore a lower energy consumption.

## 8. Conclusions

The present paper describes a numerical analysis of irreversibilities inside a heat exchanger with a heating nanofluid circulating on the shell side. The nanofluid consists of water with Al_2_O_3_ nanoparticles used for heating an aqueous medium used for oil extraction; the cold aqueous medium circulates on the tube side of the heat exchanger. The most important conclusions are:

- The nanofluid used as a heating fluid on the shell side gives off the more energy toward the aqueous medium flowing on the tube side, the larger the size of the heat exchanger.
- Nusselt numbers increase with the increase in nanofluid volume fraction, heat exchanger size, and Richardson number.
- Total entropy generation increases with the increase in nanofluid volume fraction and heat exchanger size; however, irreversibilities decrease when *Ri* ≈ 1.
- Irreversibilities due to heat transfer become more significant as the size of the heat exchanger increases.
- In general, the criterion used to evaluate the performance of the heat exchanger increases with the Richardson number when *Ri* < 1 and *Ri* > 2. On the other hand, PEC is increased by reducing the size of the heat exchanger. The nanofluid volume fraction tends to increase the performance evaluation criterion if *Ri* > 5.
- The optimal operation of heat exchangers that use aqueous media for oil extraction occurs when *H* ≥ 0.8, *φ* = 0.06 and *Ri* ≈ 1; in this range of parameters the system can operate under conditions of minimum entropy generation which implies the minimization of irreversibilities guaranteeing the efficient use of energy.
- The key achievement of this research is the characterization of the entropy minimization in heat exchangers for the aqueous extraction of oil at low temperatures, which avoids the use of solvents that cause problems in industrial safety, environmental pollution and risks for human health.
- The results obtained in this work would be beneficial in the future to improve the thermal performance of heat exchangers used in food processing, biochemical processes, solar thermal collectors and the chemical industry, guaranteeing the minimization of energy costs in such applications.
- Future research includes multiphase flows, high temperature processes and study of different nanofluids.

## Figures and Tables

**Table 1:** Comparison of Nusselt, entropy, and Bejan numbers for two different cases with five different meshes.

Nodes of the Mesh	150,240	200,175	250,645	300,240	350,422
*Ri* = 5, *H* = 0.8, *φ* = 0.04					
*Nu*	52.4558	48.2885	46.7369	46.0871	45.6941
*S*	70.6391	64.4988	61.9109	61.2252	60.7875
*Be*	0.7429	0.6861	0.6614	0.6527	0.6466
*Ri* = 0.1, *H* = 0.6, *φ* = 0.00					
*Nu*	40.9913	37.2716	36.1299	35.6981	35.3727
*S*	56.5378	51.8838	49.7830	49.1102	48.5662
*Be*	0.1995	0.1821	0.1757	0.1735	0.1721

**Table 2:** Comparison of temperatures obtained experimentally and numerically in the upper, middle and lower parts of the outer surface of the heat exchanger.

Nodes of the Mesh	Texp (°C)	T (°C)	Error
*Ri* = 5, *H* = 1.0, *φ* = 0.04			
Top	73.58	72.81	1.05%
Middle	59.17	58.66	0.87%
Bottom	38.73	38.30	1.12%
*Ri* = 5, *H* = 0.6, *φ* = 0.04			
Top	76.59	75.63	1.27%
Middle	62.14	61.57	0.93%
Bottom	42.89	42.44	1.06%

## Nomenclature

Symbol	
*Be*	Bejan number
*C_p_*	specific heat, J kg^−1^ K^−1^
*g*	acceleration of gravity, ms^−2^
*H_e_*	height of heat exchanger, m
*H*	dimensionless height of heat exchanger
*J*	vertical unit vector
*k*	thermal conductivity, W m^−1^ K^−1^
*n*	time step
*Nu*	Average Nusselt number
*p*	pressure, Pa
*PEC*	performance evaluation criterion
*Pr*	Prandtl number
*Ri*	Richardson number
*Re*	Reynolds number
*S*	total dimensionless entropy generation
*S_lf_*	dimensionless local entropy generation due to fluid friction
*S_lh_*	dimensionless local entropy generation due to heat transfer
*T*	temperature, °C
*U, V*	dimensionless transverse components of velocity
*W*	dimensionless axial component of velocity
*U_in_*	inlet velocity on the shell side, ms^−1^
*X, Y*	dimensionless transverse coordinates
*Z*	axial dimensionless coordinate
Greek Symbols	
*α*	thermal diffusivity, m^2^ s^−1^
*β*	coefficient of thermal expansion, K^−1^
*ρ*	density, kg m^−3^
*Γ*	Dirichlet boundary condition in the domain
*Ω*	three-dimensional domain
*ψ*	finite element weight function
*φ*	irreversibility ratio
*μ*	dynamic viscosity, Pa s
*θ*	dimensionless temperature
*φ*	nanofluid volume fraction
Sub-indices	
*nf*	nanofluid
*f*	base fluid
*sol*	solid
*ci*	cold fluid inlet
*hi*	hot fluid inlet
